# L-shaped relationship between water deficit and prevalence of chronic kidney disease among adults in the USA: National Health and Nutrition Examination Survey

**DOI:** 10.1017/S0007114525105667

**Published:** 2026-01-14

**Authors:** Yumiko Inoue, Daiki Watanabe, Motohiko Miyachi

**Affiliations:** 1 Graduate School of Sport Sciences, Waseda University, 2-579-15 Mikajima, Tokorozawa-city, Saitama 359-1192, Japan; 2 National Institute of Health and Nutrition, National Institutes of Biomedical Innovation, Health and Nutrition, 3-17 Senrioka Shinmachi, Settsu-city, Osaka 566-0002, Japan; 3 Faculty of Sport Sciences, Waseda University, 2-579-15 Mikajima, Tokorozawa-city, Saitama 359-1192, Japan; 4 National Institute of Biomedical Innovation, National Institutes of Biomedical Innovation, Health and Nutrition, 7-6-8 Saito-Asagi, Ibaraki-city, Osaka 567-0085, Japan

**Keywords:** Dehydration, Water deficit, Water intake, Water turnover

## Abstract

Dehydration, assessed by urine and blood evaluation, is a risk factor for chronic kidney disease (CKD). The association between water deficit, as determined by a dietary assessment, and CKD prevalence is unclear. Therefore, this study aimed to clarify this association among adults in the USA. This cross-sectional study included the data of 9332 participants aged 18 years or older from the 2009–2012 National Health and Nutrition Examination Survey. Water turnover was calculated using an equation developed by the International Doubly Labelled Water Database Group. Total water intake was assessed by 24-h dietary recall on ≥ 1 d. Water deficit was defined as the ratio of water intake to water turnover. CKD was defined as an estimated glomerular filtration rate < 60 ml/min/1·73 m^2^, calculated by the 2021 CKD Epidemiology formula. OR for CKD prevalence were calculated using multivariate logistic regression and restricted cubic spline models. The mean daily water intake, turnover and deficit were 2799 ml, 3290 ml and –15 %, respectively. CKD prevalence was 6·3 %. After adjusting for lifestyle and urine and serum osmolality, the fourth water deficit quartile was inversely associated with CKD prevalence when compared with the first quartile (OR, 0·71; 95 % CI, 0·51, 0·98). In the spline analysis, the water deficit at which the OR for CKD prevalence plateaued was approximately –30 % to 0 %. Water deficit had an L-shaped association with CKD prevalence independent of urine and serum osmolality, highlighting the importance of assessing water intake relative to dietary needs. These findings may assist the development of water requirements.

Water is a vital component of human life, and the maintenance of body water is dependent on the balance between water intake and excretion^(1)^. The hydration status is commonly assessed using blood and urine osmolality^(2)^, but the concordance of these indices has not been adequately studied. Fluid status, which is assessed by evaluating urine and blood, is important for determining dehydration in clinical practice and understanding its development.

Water turnover is considered the gold standard for evaluating body water elimination, measured using the doubly labelled water (DLW) method or deuterium dilution techniques^(3–6)^. Assuming that water elimination – primarily through urine, insensible perspiration, sweat, and feces – is in equilibrium with water intake – principally pre-formed water from foods and beverages and metabolic water generated by the oxidation of energy-yielding nutrients – water turnover reflects the daily water requirement^(4,7)^. The International DLW Database Group recently developed a predictive equation for water turnover based on DLW measurements that provide an estimate of the average water requirement, thus reflecting the biological needs of a population^(3,5)^.

Chronic kidney disease (CKD) is a progressive disease that affects > 10 % of the general population worldwide (> 800 million people), and it is one of the few non-communicable diseases associated with an increasing rate of death^(8)^. Increased plasma osmolality, which is used as an indicator of dehydration, is an independent risk factor for CKD development^(9)^. Previous studies have reported inconclusive results regarding the relationship between water intake and CKD in adults because they only assessed water intake and did not account for varying water requirements among individuals^(10–16)^. One previous study reported that water intake relative to water requirements (water deficit) has an L-shaped relationship with total mortality and CVD mortality^(17)^. However, to the best of our knowledge, the association between water deficit and CKD prevalence has not been examined. Examining data regarding water supply and water demand imbalances, including water turnover and dietary water intake, may help resolve discrepancies in the association between dietary water content and CKD.

Therefore, this study aimed to evaluate concordance between urine and blood indicators related to water status and examine the relationship between water turnover, water deficit and CKD prevalence. Although plasma osmolality is considered the main indicator of the hydration status, its validity is limited by factors associated with dehydration, such as exercise and heat stress; therefore, it may not adequately reflect preclinical water deficits^(18)^. Conversely, urinary osmolality responds to changes in water balance; therefore, it may more accurately reflect the water status under independent living conditions^(19)^. Thus, we hypothesised that urine and blood indices for dehydration do not correspond to each other. Water deficit has an L-shaped relationship with the risk of mortality^(17)^, and the health benefits of water may plateau when its intake exceeds the requirement of an individual. Therefore, we also hypothesised that an L-shaped inverse relationship exists between water deficit and CKD prevalence. Additionally, we expected that the CKD prevalence would be higher among those whose water intake does not meet their personal daily water requirements.

## Materials and methods

### Study design and population

**Figure 1.**
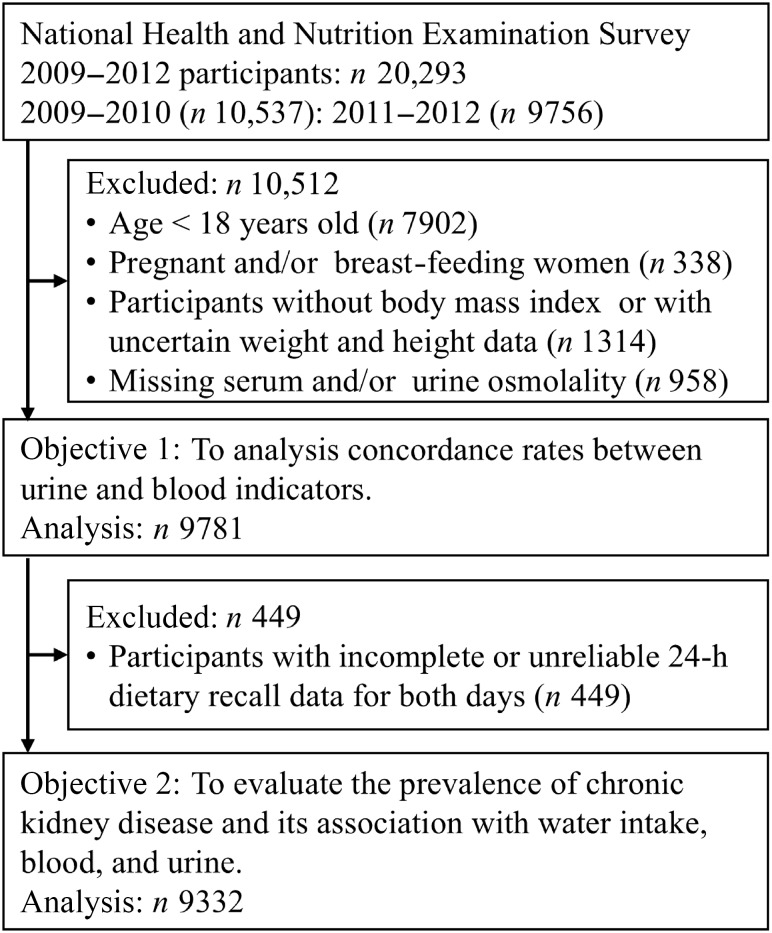
Participant flow diagram. The water deficit and chronic kidney disease prevalence according to the National Health and Nutrition Examination Survey were analysed.

This cross-sectional study used data from the USA National Health and Nutrition Examination Survey (NHANES). This recurring survey represents the non-institutionalised USA citizen population and is conducted by the National Center for Health Statistics, which is part of the Centers for Disease Control and Prevention. The methodology of the survey has been previously reported^(20)^. To reflect the USA population, the sample was selected using a complex multistage probability sampling method. This study included face-to-face household interviews, followed by health examinations performed at a mobile examination centre (MEC). We extracted electronic data from two cycles between 2009 and 2012; these data are publicly available in the National Center for Health Statistics website, and paired blood and urine samples were available^(21)^. Of the 2009–2012 NHANES participants (*n* 20 923), we excluded those younger than 18 years of age (*n* 7902), pregnant or lactating women (*n* 338), those without accurate height and weight data (*n* 1314) and those with deficient serum or urine osmolality (*n* 958). Initially, 9781 participants (4778 women and 5003 men) were included this study. After excluding individuals who provided dietary data deemed unreliable by trained NHANES interviewers and those with missing 24-h dietary recall (24HDR) information (*n* 449), 9332 participants (4546 women and 4786 men) were included in the analysis (Figure 1).

### Dietary assessment and total water intake estimation

Dietary intake was assessed by trained interviewers using 24HDR for at least 1 d. Ninety percent of participants provided 2 d of 24HDRs. The data were collected using the USA Department of Agriculture automated multiple-pass method, which is an automated five-step approach^(22)^. Participants were asked to report all food and beverages consumed within the previous 24 h (from midnight to midnight). Dietary intake was calculated using the USA Department of Agriculture Food and Nutrition Database for Dietary Studies for all reported food and beverages^(23)^. Total water intake was assessed using the total water content (ml) of all food, beverages, and plain water was included in the 24HDR information.

### Estimating water turnover

Water turnover was estimated using a formula previously established by the International DLW Database Group. This formula is based on a multiple regression model in which water turnover measured by the DLW method served as the dependent variable, and selected environmental and lifestyle factors were included as predictors^(3)^. This model was developed to predict water turnover in adults older than 18 years of age included in the international DLW database. The coefficient of determination (R^2^) for this model was 0·471, and the following equation was used:
(1)

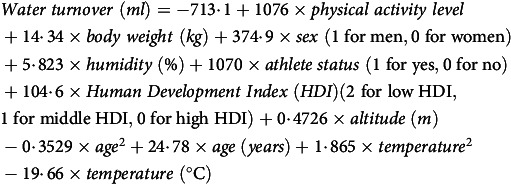




In this study, the physical activity level was calculated by dividing the calibrated energy intake by the basal metabolic rate. Calibrated energy intake was calculated using an equation developed through stepwise multiple regression analysis, with total energy expenditure measured by the DLW method as the dependent variable, and age, sex, BMI and energy intake as independent variables (R^2^ = 0·36)^(24)^. The equation used to determine the calibrated energy intake was developed to reduce systematic errors in food frequency questionnaires, and its validity was verified using repeated measurements of total energy expenditure measured by the DLW method in the same group from whom the equation was developed (Spearman’s rank correlation coefficient = 0·517)^(25)^. Its external validity was also supported by a recent analysis using data extracted from multiple DLW studies^(26)^. The basal metabolic rate was calculated using the Schofield formula^(27)^. Because the equation used to predict water turnover includes the physical activity level derived from the DLW method, we believe that dividing the calibrated energy intake by the basal metabolic rate provides a more accurate estimation of the physical activity level. Height and weight were measured by trained medical personnel using standardised procedures and calibrated equipment^(28)^. Regarding the athlete status, all participants were uniformly assessed as non-athletes, and the Human Development Index was set to high in accordance with a previous study^(3)^. When data regarding the place of residence were not available, USA altitude was estimated as 760 m based on information from *The World Factbook* ^(29)^. Temperature and relative humidity were calculated based on data from the National Oceanic and Atmospheric Administration USA Climate Reference Network weather station^(30)^ and the Met Office climate dashboard^(31)^ (2009–2010: temperature, 11·1°C; relative humidity, 66·3 %; 2011–2012: temperature, 11·0°C; relative humidity, 66·3 %).

To calculate an individual’s water balance (the difference between the total water intake and the water requirement), the water deficit was calculated using the following formula^(17)^:

Water deficit (%) = (dietary total water intake [ml/d] – water turnover [ml/d])/water turnover [ml/d] × 100.

### Blood testing

Blood samples were obtained to measure serum osmolality, blood urea nitrogen (BUN) and creatinine (Cr). First, venipuncture was performed at the MEC to collect blood in EDTA tubes following standard protocols. Then, blood was centrifuged (2900 × ***g*** at 4–8°C for 10 min) using a DxC800 system (Beckman Coulter, Brea, CA, USA) to obtain plasma. The Na^+^ concentration was measured using an indirect (or dilute) ion-selective electrode method. The Cr level was measured using the Jaffe velocity method (dynamic alkaline picric acid). The glucose concentration was quantitatively measured in serum using the oxygen rate method (glucose oxidase method) and a Beckman oxygen electrode. The BUN concentration was quantitatively measured in serum using the enzyme conductivity method. Next, serum osmolality was calculated based on Na^+^, glucose, and BUN concentrations using the following equation:

1·86 × [Na^+^ (mmol/l)] + glucose (mg/dl)/18 + BUN (mg/dl)/2·8 + 9.

Detailed methods are described elsewhere^(32)^. Serum osmolality > 295 mOsm/kg^(33)^ and BUN/Cr > 20^(34)^ indicated dehydration.

### Urine testing

Urine samples were collected at the MEC and used to measure osmolality and flow rate. Urine osmolality was measured using an Osmette II Model 5005 automatic osmometer (Precision Systems Inc.) and the freezing point depression method. The urine flow rate was calculated by dividing the total urine volume by total voiding time. Participants recorded the time of their last void before providing a urine sample at the MEC and the time when they provided their urine sample at the MEC. If the initial sample was insufficient, then up to two additional urine samples were collected and the volume and time of each collection were recorded. Finally, these samples were combined and the urine flow rate was calculated. Dehydration was defined as urine osmolality > 700 mOsm/kg^(35,36)^ and a urine flow rate of 0 to 0·5 ml/min (first quartile)^(37)^.

### Definition of chronic kidney disease

CKD was defined according to previously published descriptions^(38)^. The estimated glomerular filtration rate (eGFR) was calculated from sex, age and serum Cr using the CKD Epidemiology (CKD-EPI) 2021 equation^(39)^. An eGFR < 60 ml/min/1·73 m^2^ indicated CKD^(40)^.

### Statistical analysis

The total water intake, water turnover, water deficit, serum osmolality, urine osmolality and urine flow rate were classified into four groups according to their respective quartiles. Participant characteristics are described as the mean and standard deviation for continuous variables and number and percentage for categorical variables. However, the following variables were missing: education (*n* 10); smoking status (*n* 262); alcohol consumption (*n* 847); marital status (*n* 472); poverty-to-income ratio (*n* 778); medication use (*n* 4); history of hypertension (*n* 13); history of diabetes (*n* 5) and physical activity (*n* 29). Indices were created to impute these missing values.

Spearman’s rank correlation coefficient was calculated to compare blood and urine dehydration indices. The kappa statistic was used to validate the concordance rates of individuals with dehydration, as determined by the blood and urine indices.

Multivariate logistic regression models were used to evaluate the association between total water intake, urinary and blood indices and CKD prevalence. Results of the analyses are presented as OR and 95 % CI of the first quartile. A restricted cubic spline model with three sections (10th, 50th and 90th percentiles), following the distributions of exposure variables, was used to determine the dose–response relationship between total water intake and OR for urinary blood indices and CKD prevalence. Because data were sparse, the analysis was discontinued at the 99th percentile of the total water intake, water turnover, water deficit, urine osmolality and urine flow distributions in the spline model, as well as at the 1st and 99th percentiles of the serum osmolality distribution^(17)^. The *P* values for linear trends were calculated by treating the exposure variables as continuous variables. Statistical dominance of non-linearity was assessed using the Wald test to compare the likelihood ratios of the spline and linear models; *P* < 0·05 indicated a statistically significant non-linear relationship between exposure and outcome^(41)^.

After performing multiple imputation to handle missing covariate data, sensitivity analyses were conducted. Variables with missing values, including education, smoking status, alcohol consumption, marital status, poverty-to-income ratio, medication use, history of hypertension, history of diabetes and physical activity were imputed using chained equations with logistic regression models to generate 20 datasets. Variables without missing values, including age and energy intake included in the multivariable model, were used as predictors in the imputation models, and a random seed was set to ensure reproducibility. The 20 imputed datasets were analysed using the ‘mi Estimate’ command in STATA MP version 18.0 (StataCorp LP), and the results were pooled. All missing values were assumed to be missing at random.

Multivariate analyses were performed using previously reported models including potential confounders^(10,37)^. Model 1 was adjusted for age (continuous), sex (female/male) and race/ethnicity (Mexican American, other Hispanic, non-Hispanic White, non-Hispanic Black or others). Model 2 was adjusted for all variables in model 1 as well as BMI (continuous), education level (less than high school, high school or equivalent, and more than high school), smoking status (never, ever, or currently), alcohol consumption (never, ever or currently), marital status (married, widowed, divorced, separated, never married or living with a partner), poverty-to-income ratio (< 1·3, 1·3–3·5, ≥ 3·5), medication use (yes/no), history of hypertension (yes/no), history of diabetes (yes/no), energy intake (continuous) and physical activity level (sedentary, insufficient, moderate or high). Weekly metabolic equivalents (MET) were calculated by multiplying the NHANES-recommended metabolic equivalent value by daily activity (in min) and activity per week (in days)^(42)^. Leisure time, occupation/household and transportation-related physical activity were assessed, and each respondent was classified into one of the following four categories: sedentary (< 10 min/week); insufficient activity (10–149 min/week); moderate activity (150–300 min/week) or high activity (≥ 300 min/week). To examine whether the association between total water intake or water deficit and CKD was independent of blood and urine indicators of dehydration, Model 3 included the variables in Model 2 total plus water intake, water turnover and water deficit, with adjustment for serum and urine osmolality. For analyses of serum osmolality, urine osmolality and urine flow rate, adjustments were for total water intake and water turnover.

All statistical analyses were conducted using a two-sided significance level < 5 %. All analyses were performed using STATA MP version 18.0 (StataCorp LP).

## Results

Table 1 presents the participants’ characteristics across water deficit quartiles. Participants with higher water deficits were more likely to be male, to smoke and consume alcohol and to have a higher educational level. Moreover, they had lower prevalences of hypertension and diabetes and tended to have a lower BMI. Serum osmolality was weakly correlated with BUN/Cr, urine osmolality and urine flow; however, dehydration classification based on these indices did not show agreement (Table 2). Water deficit showed significant correlations with blood and urine indices; however, these correlations were not considered meaningful (online Supplementary Table 1).

**Table 1.** Participant characteristics according to water deficit quartiles (Mean values and standard deviations; numbers and percentages)

Variable	Water deficit quartile									
	Total (*n* 9332)		Q1 (*n* 2333)		Q2 (*n* 2333)		Q3 (*n* 2333)		Q4 (*n* 2333)	
	Mean	sd	Mean	sd	Mean	sd	Mean	sd	Mean	sd
Age (years)^*^	46·9	17·9	46·5	18·9	48·0	18·2	47·9	17·5	45·1	16·9
	*n*	%	*n*	%	*n*	%	*n*	%	*n*	%
Women (*n*, %)^†^	4546	48·7	1183	50·7	1174	50·3	1107	47·5	1082	46·4
	Mean	sd	Mean	sd	Mean	sd	Mean	sd	Mean	sd
Height (cm)^*^	167·9	10·1	166·6	10·1	167·0	10·2	168·4	10·1	169·4	10·0
Body weight (kg)^*^	81·1	20·8	81·3	21·9	81·3	21·2	81·6	20·6	80·4	19·2
BMI (kg/m^2^)^*^	28·7	6·5	29·2	7·1	29·0	6·8	28·7	6·3	27·9	5·9
	*n*	%	*n*	%	*n*	%	*n*	%	*n*	%
Non-Hispanic, white (*n*, %)^†^	4030	43·2	707	30·3	949	40·7	1108	47·5	1266	54·3
Education, college or higher (*n*, %)^†,^^‡^	4834	51·8	968	41·5	1185	50·8	1185	50·8	1395	59·8
Current smoker (*n*, %)^†,^^‡^	2036	21·8	479	20·5	471	20·2	482	20·7	604	25·9
Current drinker (*n*, %)^†,^^‡^	6346	68·0	1371	58·8	1559	66·8	1636	70·1	1780	76·3
Marital status, married (*n*, %)^†,^^‡^	4503	48·3	1046	44·8	1133	48·6	1191	51·1	1133	48·6
	Mean	sd	Mean	sd	Mean	sd	Mean	sd	Mean	sd
Poverty-to-income ratio^*,^^‡^	2·4	1·7	2·1	1·6	2·5	1·6	2·6	1·7	2·7	1·7
Blood urea nitrogen (mg/dl)^*^	12	5	13	6	13	6	13	5	12	5
Cr (mg/dl)^*^	0·90	0·37	0·92	0·52	0·89	0·37	0·89	0·33	0·87	0·21
BUN/Cr^*^	14·9	5·1	14·6	5·1	15·2	5·3	15·0	5·1	14·6	4·9
eGFR (ml/min/1·73 m^2^)^*^	97	22	96	24	96	22	96	22	99	20
Serum osmolality (mOsm/kg)^*^	278	5	278	5	278	5	278	5	277	5
Urine osmolality (mOsm/kg)^*^	629	267	695	258	655	253	603	264	565	272
Urine flow rate (ml/kg/h)^*,^^‡^	1·05	0·95	0·84	0·80	0·92	0·78	1·11	0·98	1·31	1·14
	*n*	%	*n*	%	*n*	%	*n*	%	*n*	%
Medication used (*n*, %)^†,^^‡^	5076	54·4	1236	53·0	1315	56·4	1308	56·1	1217	52·2
Hypertension (*n*, %)^†,^^‡^	3050	32·7	803	34·4	793	34·0	787	33·7	667	28·6
Diabetes mellitus (*n*, %)^†,^^‡^	994	10·7	276	11·8	262	11·2	243	10·4	213	9·1
	Mean	sd	Mean	sd	Mean	sd	Mean	sd	Mean	sd
Energy intake (kJ/d)^*^	8872	3598	7217	2728	8485	3314	9192	3498	10 033	4247
Calibrated energy intake (kJ/d)^*^	10 184	1510	9841	1339	10 134	1448	10 305	1519	10 456	1657
Basal metabolic rate (kJ/d)^*^	6853	1322	6812	1360	6803	1305	6883	1318	6916	1301
Physical activity level^*,^^‡^	1·51	0·17	1·47	0·16	1·51	0·16	1·52	0·17	1·53	0·18
Total water intake (ml/d)^*^	2799	1278	1554	361	2270	323	2949	426	4421	1279
Water turnover (ml/d)^*^	3290	397	3235	380	3278	392	3311	402	3339	406
Water deficit (%)^*^	–15·4	35·7	–52·0	9·3	–30·7	5·2	–10·9	6·6	32·2	33·1

BUN, blood urea nitrogen; Cr, creatinine; eGFR, estimated glomerular filtration rate; SD, standard deviation; Q, quartile.

Water deficit is defined as the total water intake minus the water turnover divided by the water turnover.

BMI is defined as weight (kg) divided by height squared (m^2^).

Q1 to Q4 correspond to water deficits of < –39·7, –39·7 to –21·3, –21·4 to 1·2, and > 1·3, respectively.

* Continuous values are shown as means (SD).

† Categorical values are shown as numbers (percentages).

‡ Missing data were as follows: education status, 10 participants; smoking status, 262 participants; alcohol consumption, 847 participants; marital status, 472 participants; poverty-to-income ratio, 778 participants; medication use, 4 participants; history of hypertension, 13 participants; history of diabetes, 5 participants and physical activity, 29 participants.

**Table 2.** Prevalence and concordance of dehydration defined by blood and urine indices (Mean values and standard deviations; percentages and 95 % confidence intervals)

	Mean	sd	*r* ^†^	Prevalence of dehydration^‡^			Kappa statistic^§^	95 % CI
				Case	%	95 % CI		
Total (*n* 9781)								
Serum osmolality (mOsm/kg)	278	5	Ref	26	0·3	0·2, 0·4	Ref	
BUN/Cr	14·9	5·2	0·30^*^	1629	16·7	15·9, 17·4	0·01	0·00, 0·01
Urine osmolality (mOsm/kg)	630	267	0·14^*^	4329	44·3	43·3, 45·2	0·00	–0·01, 0·00
Urine flow rate (ml/min)	1·0	1·1	–0·12^*^	2322	23·7	22·9, 24·6	0·00	0·00, 0·00
Men (*n* 5003)								
Serum osmolality (mOsm/kg)	279	5	Ref	11	0·2	0·1, 0·4	Ref	
BUN/Cr	13·7	4·3	0·30^*^	482	9·6	8·8, 10·5	0·02	0·01, 0·02
Urine osmolality (mOsm/kg)	668	258	0·19^*^	2495	49·9	48·5, 51·3	0·00	–0·01, 0·00
Urine flow rate (ml/min)	1·1	0·9	–0·12^*^	1018	20·4	19·3, 21·5	0·00	–0·01, 0·00
Women (*n* 4778)								
Serum osmolality (mOsm/kg)	278	5	Ref	15	0·3	0·2, 0·5	Ref	
BUN/Cr	16·2	5·7	0·36^*^	1147	24·0	22·8, 25·2	0·00	0·00, 0·00
Urine osmolality (mOsm/kg)	590	269	0·10^*^	1834	38·4	37.0, 39·8	0·00	0·00, 0·01
Urine flow rate (ml/min)	1·0	1·3	–0·14^*^	1304	27·3	26.0, 28·6	–0·01	–0·01, 0·00

BUN, blood urea nitrogen; Cr, creatinine; SD, standard deviation; Q, quartile; Ref, reference.

* Indicates statistical significance of Spearman’s rank correlation coefficient (*P* < 0·05).

† The statistical analysis was performed using Spearman’s rank correlation coefficient to compare individual rankings determined by serum osmolality and blood and urine indices.

‡ Dehydration was evaluated separately according to the following criteria: serum osmolality > 295 mOsm/kg, BUN/Cr > 20, urine osmolality > 700 mOsm/kg and urine flow rate of 0–0·5 ml/min (Q1).

§ Agreement between dehydration assessed according to BUN/Cr, urine osmolality and urine flow rate and dehydration assessed according to serum osmolality was evaluated. The kappa statistic ranged from –1 (perfect disagreement) to 1 (perfect agreement), with 0 indicating agreement attributable to chance.

Table 3 shows the association between total water intake, dehydration indices and CKD prevalence. The CKD prevalence in this study was 6·3 % (95 % CI, 5·8, 6·8). After adjusting for lifestyle factors and urine and serum osmolality, the fourth quartiles of water deficit and total water intake were inversely associated with CKD prevalence when compared with the first quartile (water deficit: OR, 0·71; 95 % CI, 0·51, 0·98; total water intake: OR, 0·63; 95 % CI, 0·44, 0·91). Serum osmolality was positively associated with CKD prevalence (quartile 4 (Q4): OR, 4·41; 95 % CI, 3·07, 6·34), whereas urine osmolality (Q4: OR, 0·50; 95 % CI, 0·33, 0·76) and the urine flow rate (Q4: OR, 0·59; 95 % CI, 0·43, 0·80) were inversely associated with CKD prevalence. Similar results were obtained by performing a sensitivity analysis (online Supplementary Table 2). Water deficit and total water intake were inversely associated with dehydration prevalence, as determined by urine osmolality, but not with serum osmolality (Table 4).

**Table 3.** Odds ratios for chronic kidney disease according to the total water intake quartile and blood and urine indicators (Numbers and percentages; mean values and standard deviations; odds ratios and 95 % confidence intervals)

	Case			Mean	sd	Model 1^*^		Model 2^†^		Model 3^‡^	
	*n*	%				OR	95 % CI	OR	95 % CI	OR	95 % CI
Total water intake^§^											
Q1 (*n* 2333)	237	10·2		1510	309	1·00	Ref	1·00	Ref	1·00	Ref
Q2 (*n* 2333)	164	7·0		2238	184	0·84	0·66, 1·07	0·85	0·66, 1·09	0·80	0·62, 1·04
Q3 (*n* 2333)	129	5·5		2941	232	0·81	0·63, 1·04	0·84	0·63, 1·10	0·78	0·58, 1·04
Q4 (*n* 2333)	58	2·5		4505	1203	0·58	0·42, 0·81	0·61	0·43, 0·87	0·63	0·44, 0·91
* P* for trend						0·001		0·005		0·007	
* P* for non-linearity						0·349		0·349		0·362	
Water turnover^§^											
Q1 (*n* 2333)	286		12·3	2784	206	1·00	Ref	1·00	Ref	1·00	Ref
Q2 (*n* 2333)	131		5·6	3154	94	0·89	0·68, 1·17	0·67	0·49, 0·93	0·70	0·50, 0·98
Q3 (*n* 2333)	109		4·7	3447	73	1·30	0·91, 1·85	0·87	0·57, 1·34	1·02	0·66, 1·60
Q4 (*n* 2333)	62		2·7	3777	193	1·18	0·77, 1·83	0·70	0·38, 1·27	0·91	0·49, 1·71
* P* for trend						0·297		0·005		0·004	
* P* for non-linearity						0·206		0·464		0·132	
Water deficit^§^											
Q1 (*n* 2333)	198		8·5	–52	9	1·00	Ref	1·00	Ref	1·00	Ref
Q2 (*n* 2333)	154		6·6	–31	5	0·75	0·58, 0·96	0·77	0·59, 0·99	0·75	0·57, 0·98
Q3 (*n* 2333)	143		6·1	–11	7	0·74	0·57, 0·95	0·81	0·61, 1·06	0·75	0·56, 0·99
Q4 (*n* 2333)	93		4·0	32	33	0·59	0·44, 0·79	0·69	0·50, 0·94	0·71	0·51, 0·98
* P* for trend						< 0·001		0·022		0·031	
* P* for non-linearity						0·296		0·512		0·271	
Serum osmolality^§^											
Q1 (*n* 2662)	40		1·5	273	3	1·00	Ref	1·00	Ref	1·00	Ref
Q2 (*n* 2499)	85		3·4	277	1	2·00	1·34, 2·99	1·98	1·32, 2·98	1·98	1·31, 2·98
Q3 (*n* 2179)	110		5·0	280	1	1·99	1·35, 2·94	1·90	1·28, 2·82	1·88	1·27, 2·80
Q4 (*n* 1992)	353		17·7	284	3	5·04	3·54, 7·17	4·42	3·09, 6·33	4·41	3·07, 6·34
* P* for trend						< 0·001		< 0·001		< 0·001	
* P* for non-linearity						< 0·001		0·003		0·005	
Urine osmolality^§^											
Q1 (*n* 2339)	153		6·5	270	87	1·00	Ref	1·00	Ref	1·00	Ref
Q2 (*n* 2338)	284		12·1	540	70	1·35	1·07, 1·70	1·23	0·97, 1·56	1·18	0·92, 1·50
Q3 (*n* 2338)	117		5·0	750	53	0·73	0·55, 0·96	0·69	0·52, 0·92	0·65	0·48, 0·86
Q4 (*n* 2317)	34		1·5	961	87	0·53	0·35, 0·80	0·56	0·37, 0·85	0·50	0·33, 0·76
* P* for trend						0·001		< 0·001		< 0·001	
* P* for non-linearity						< 0·001		< 0·001		< 0·001	
Urine flow rate^§^											
Q1 (*n* 2217)	215		9·7	0·3	0·1	1·00	Ref	1·00	Ref	1·00	Ref
Q2 (*n* 2210)	136		6·2	0·6	0·1	0·75	0·58, 0·96	0·57	0·57, 0·96	0·76	0·57, 0·97
Q3 (*n* 2213)	121		5·5	1·0	0·1	0·76	0·58, 0·99	0·58	0·58, 0·99	0·78	0·59, 1·03
Q4 (*n* 2211)	75		3·4	2·2	0·1	0·53	0·39, 0·73	0·40	0·40, 0·75	0·59	0·43, 0·80
* P* for trend						< 0·001		< 0·001		0·001	
* P* for non-linearity						0·229		0·217		0·300	

Q, quartile; Ref, reference.

* Model 1: adjusted for age, sex and race/ethnicity.

† Model 2: adjusted for all factors listed in model 1 as well as BMI, education, smoking status, alcohol consumption, marital status, poverty-to-income ratio, medication use, history of hypertension, history of diabetes mellitus, energy intake and physical activity.

‡ Model 3: adjusted for all factors listed in model 2 as well as total water intake, water turnover and water deficit, which were adjusted for serum and urine osmolality. Serum osmolality, urine osmolality and urine flow rate were adjusted for total water intake and water turnover.

§ Total water intake quartiles are as follows: Q1, < 1928; Q2, 1928–2572; Q3, 2573–3389 and Q4, ≥ 3390 ml/d. Water turnover quartiles are as follows: Q1, < 2999; Q2, 2999–3312; Q3, 3313–3570 and Q4, ≥ 3571 ml/d. Water deficit quartiles are as follows: Q1, < –39·7; Q2, –39·7 to –21·3; Q3, –21·4 to 1·2 and Q4, ≥ 1·3 %. Serum osmolality quartiles are as follows: Q1, < 276; Q2, 276–278; Q3, 279–281 and Q4, ≥ 282 mOsm/kg. Urine osmolality quartiles are as follows: Q1, < 410; Q2, 410–655; Q3, 656–840 and Q4, ≥ 841 mOsm/kg. Urine flow rate quartiles are as follows: Q1, < 0·5; Q2, 0·5–0·7; Q3, 0·8–1·2 and Q4, ≥ 1·3 ml/min.

**Table 4.** Odds ratios of dehydration assessed using blood and urine indicators according to total water intake, water turnover, and water deficit quartiles (Numbers and percentages; mean values and standard deviations; odds ratios and 95 % confidence intervals)

	Case		Mean	sd	Model 1^*^		Model 2^†^		Model 3^‡^	
	*n*	%			OR	95 % CI	OR	95 % CI	OR	95 % CI
Total water intake^§^										
Serum osmolality for dehydration^||^										
Q1 (*n* 2333)	12	0·5	1510	309	1·00	Ref	1·00	Ref	1·00	Ref
Q2 (*n* 2333)	4	0·2	2238	184	0·45	0·14, 1·41	0·48	0·15, 1·58	0·43	0·13, 1·44
Q3 (*n* 2333)	6	0·3	2941	232	0·86	0·32, 2·37	0·84	0·28, 2·50	0·58	0·19, 1·79
Q4 (*n* 2333)	1	0	4505	1203	0·21	0·03, 1·70	0·23	0·03, 1·97	0·15	0·02, 1·32
* P*_for trend_					0·269		0·388		0·157	
* P*_for non-linearity_					0·349		0·349		0·362	
Urine osmolality for dehydration^||^										
Q1 (*n* 2333)	1128	48·3	1510	309	1·00	Ref	1·00	Ref	1·00	Ref
Q2 (*n* 2333)	1089	46·7	2238	184	0·91	0·80, 1·02	0·78	0·68, 0·88	0·78	0·69, 0·89
Q3 (*n* 2333)	967	41·4	2941	232	0·68	0·60, 0·77	0·53	0·47, 0·61	0·55	0·48, 0·62
Q4 (*n* 2333)	967	41·4	4505	1203	0·55	0·48, 0·62	0·38	0·33, 0·44	0·40	0·35, 0·46
* P*_for trend_					< 0·001		< 0·001		< 0·001	
* P*_for non-linearity_					0·023		< 0·001		< 0·001	
Water turnover^§^										
Serum osmolality for dehydration^§^										
Q1 (*n* 2333)	11	0·5	2784	206	1·00	Ref	1·00	Ref	1·00	Ref
Q2 (*n* 2333)	7	0·3	3154	94	0·84	0·29, 2·43	0·62	0·18, 2·13	0·66	0·19, 2·35
Q3 (*n* 2333)	2	0·1	3447	73	0·28	0·05, 1·67	0·16	0·02, 1·18	0·13	0·02, 1·04
Q4 (*n* 2333)	3	0·1	3777	193	0·51	0·09, 2·90	0·26	0·02, 3·34	0·25	0·02, 3·44
* P*_for trend_					0·292		0·005		0·003	
* P*_for non-linearity_					0·947		0·961		0·978	
Urine osmolality for dehydration^||^										
Q1 (*n* 2333)	687	29·4	2784	206	1·00	Ref	1·00	Ref	1·00	Ref
Q2 (*n* 2333)	991	42·5	3154	94	1·53	1·35, 1·74	1·23	1·06, 1·42	1·29	1·11, 1·50
Q3 (*n* 2333)	1149	49·2	3447	73	1·80	1·54, 2·10	1·21	0·99, 1·48	1·34	1·09, 1·64
Q4 (*n* 2333)	1299	55·7	3777	193	2·26	1·91, 2·67	1·20	0·92, 1·57	1·41	1·07, 1·85
* P*_for trend_					< 0·001		0·009		< 0·001	
* P*_for non-linearity_					0·130		0·042		0·073	
Water deficit^§^										
Serum osmolality for dehydration^||^										
Q1 (*n* 2333)	7	0·3	–52	9	1·00	Ref	1·00	Ref	1·00	Ref
Q2 (*n* 2333)	7	0·3	–31	5	1·20	0·41, 3·45	1·55	0·52, 4·69	1·51	0·49, 4·60
Q3 (*n* 2333)	6	0·3	–11	7	1·21	0·40, 3·68	1·47	0·46, 4·71	1·06	0·32, 3·50
Q4 (*n* 2333)	3	0·1	32	33	0·81	0·20, 3·21	1·06	0·25, 4·51	0·75	0·17, 3·26
* P*_for trend_					0·369		0·584		0·272	
* P*_for non-linearity_					0·342		0·244		0·261	
Urine osmolality for dehydration^||^										
Q1 (*n* 2333)	1254	53·8	–52	9	1·00	Ref	1·00	Ref	1·00	Ref
Q2 (*n* 2333)	1117	47·9	–31	5	0·83	0·73, 0·93	0·76	0·67, 0·86	0·76	0·67, 0·87
Q3 (*n* 2333)	926	39·7	–11	7	0·59	0·52, 0·66	0·51	0·45, 0·58	0·51	0·45, 0·59
Q4 (*n* 2333)	829	35·5	32	33	0·45	0·40, 0·51	0·38	0·33, 0·43	0·39	0·34, 0·45
* P*_for trend_					< 0·001		< 0·001		< 0·001	
* P*_for non-linearity_					0·026		< 0·001		< 0·001	

SD, standard deviation; Q, quartile; Ref, reference.

* Model 1: adjusted for age, sex and race/ethnicity.

† Model 2: adjusted for all factors listed in model 1 as well as BMI, education, smoking status, alcohol consumption, marital status, poverty-to-income ratio, medication use, history of hypertension, history of diabetes mellitus, energy intake and physical activity.

‡ Model 3: adjusted for all factors listed in model 2 as well as serum osmolality dehydration, which was adjusted for urine osmolality, and urine osmolality dehydration, which was adjusted for serum osmolality.

§ Total water intake quartiles are as follows: Q1, < 1928; Q2, 1928–2572; Q3, 2573–3389 and Q4, ≥ 3390 ml/d. Water turnover quartiles are as follows: Q1, < 2999; Q2, 2999–3312; Q3, 3313–3570 and Q4, ≥ 3571 ml/d. Water deficit quartiles are as follows: Q1, –39·7; Q2, –39·7 to –21·3; Q3, –21·4 to 1·2 and Q4, ≥ 1·3 %.

|| Dehydration was defined as serum osmolality of 295 mOsm/kg, urine osmolality > 700 mOsm/kg (Q1).

**Figure 2.**
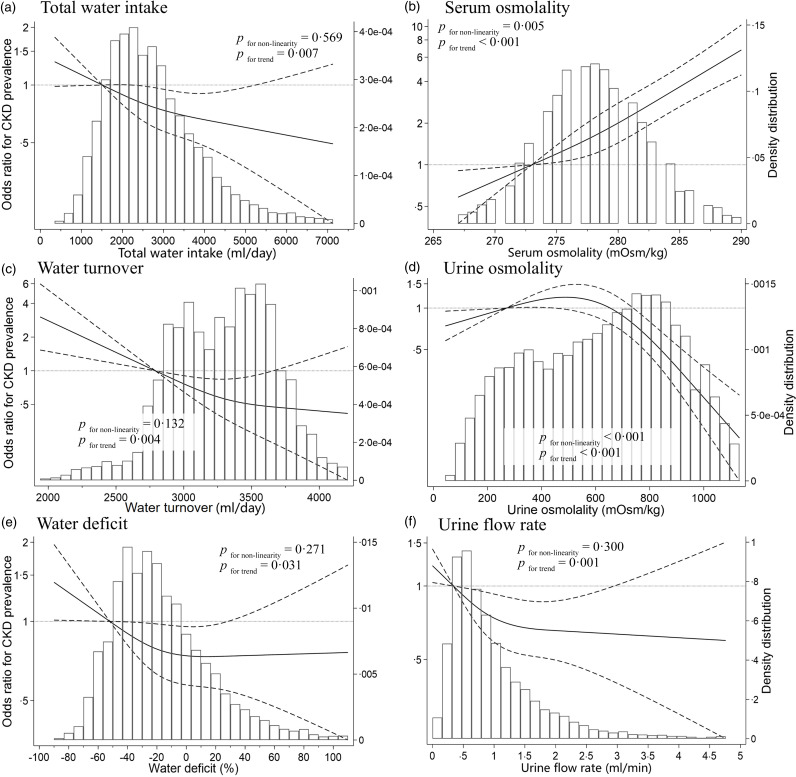
Restricted cubic spline curves of chronic kidney disease (CKD) according to water intake, blood indices and urine indices according to the National Health and Nutrition Examination Survey 2009–2012. OR according to the multivariate-adjusted logistic regression models using restricted cubic spline curves describing associations between (a) total water intake (*n* 9333), (b) serum osmolality (*n* 9333), (c) water turnover (*n* 9333), (d) urine osmolality (*n* 9333), (e) water deficit (*n* 9333) and (f) urine flow rate (*n* 8851) and CKD (vertical axis). OR are based on model 3, which was adjusted for age, sex, race/ethnicity, BMI, education, smoking status, alcohol consumption, marital status, poverty-to-income ratio, medication use, history of diabetes, history of hypertension, energy intake, physical activity, serum osmolality and urine osmolality (a, c and e) and for total water intake and water turnover (b, d, and f). OR were used to compare the reference values (horizontal axis) and mean values of participants in the first quartile (total water intake, 1510 ml; water turnover, 2784 ml; water deficit, –52 %; serum osmolality, 273 mOsm/kg; urine osmolality, 270 mOsm/kg; urine flow rate, 0·3 ml/min). Solid lines represent OR. Dashed lines represent 95 % confidence intervals.

Figure 2 shows the dose–response relationship between total water intake, dehydration indices and CKD using a restricted cubic spline model. The water deficit and urine flow rate at which the OR for CKD prevalence plateaued were approximately –30 % to 0 % and 0·9 to 1·6 ml/min, respectively. The spline analysis model of the other indices yielded results similar to those of the linear regression analysis.

## Discussion

During this study, urine and blood dehydration indices were not concordant, and water deficit exhibited an L-shaped relationship with CKD prevalence, even after adjusting for urine and blood indices that indicated dehydration. Our results indicated that the water deficit at which the OR for CKD prevalence plateaued was approximately –30 % to 0 %. To our knowledge, this is the first study to evaluate the association between CKD prevalence and water deficit, calculated using water turnover estimated from predictive equations and total water intake estimated from dietary assessments.

In the present study, the dehydration indices assessed using blood and urine samples were discordant. A previous study of independent adults found no difference in the plasma osmolality of groups with low and high water intake as assessed by dietary records; however, urinary markers of the low water intake group were higher than those of the high water intake group^(19)^. Studies of restricted water intake reported changes in dehydration indices, including decreased urine volume and increased urine specific gravity; however, plasma osmolality remained unchanged^(43)^. The kidneys regulate water balance by adjusting the urine concentration to help maintain plasma osmolality within a stable range^(44)^. Although plasma osmolality can increase during acute dehydration, it remains constant during mild dehydration with normal water intake^(45)^. Therefore, it is reasonable to believe that dehydration determined based on urine osmolality can differ from dehydration determined based on serum osmolality. However, spot urine samples, such as those used during this study, can fluctuate quickly because of factors such as exercise, diet and ambient temperature, thus limiting the information regarding the body’s daily water status compared to that provided by 24-h urine collection^(46)^. Thus, further research is necessary to determine whether dehydration assessed by the 24-h urine collection findings is related to dehydration assessed by serum osmolality.

The mean total water intake, water turnover and water deficit of our population were 2799 ml, 3290 ml and –15 %, respectively. The International DLW Database Group found that water turnover, as measured using the DLW method, ranged from 2·8 to 3·3 l/d for women aged 40–80 years and 3·1 to 4·0 l/d for men^(3)^. Similarly, a study of 458 independent individuals aged 40–79 years in the USA indicated that water turnover ranged from 2·8 to 3·3 l/d for women and 3·4 to 3·8 l/d for men^(4)^. The recommended daily water intake according to the USA and Canada guidelines^(45)^ and the WHO^(47)^ is 2·7 l/d for women and 3·2 to 3·7 l/d for men. The findings of the previous studies are consistent with those of the present study. Water intake from dietary sources such as food and beverages accounted for approximately 85 % of the water turnover, whereas other water intake, including metabolic water, accounted for approximately 10 % to 15 %^(4)^. These findings align with the water deficit at which CKD prevalence plateaus, as observed during this study. However, the 24HDR method underestimates the approximate water intake by 20 %^(48)^ and the dietary record by 50 %^(6,49)^ compared with the water intake estimated using the stable isotope method. These findings suggest that the absolute water deficit values should be interpreted with caution because water intake determined using self-reported dietary assessments may be underestimated. Such underestimates are particularly pronounced with higher water intake levels^(3)^ and may lead to underestimation of the plateau in the spline analysis. Therefore, objective methods that do not rely on self-reported dietary assessments to evaluate water intake and requirements are crucial. Our results may contribute to establish individual water requirements and relative water intake targets that allow disease prevention in the USA population.

Our findings showed that the OR for CKD prevalence plateaued at a water deficit of approximately –30 % to 0 %, indicating an L-shaped association. Cross-sectional studies reported lower OR for CKD with higher fluid intake^(10–13)^; however, inconsistent associations between fluid intake and renal function have been observed during randomised controlled trials^(15,16)^. Two factors may explain these discrepancies. First, the beneficial effects of water intake may be related to the renal function of the target population. For instance, a study of an educational intervention to promote water intake by USA adults with stage 3 CKD observed increased water intake but no reno-protective effects such as improved albuminuria levels or eGFR^(15)^. However, water intake can slow the annual rate of decline of the eGFR of patients with stage 1 or 2 CKD and preserved renal function^(16)^. Additionally, a prospective cohort study reported a U-shaped relationship between water intake and end-stage renal disease development^(14)^. Therefore, the beneficial effects of water intake may not be observed in populations with compromised renal function or those with end-stage renal disease events that are more advanced than CKD onset. Because this study evaluated the association between water intake and CKD prevalence in a general healthy population, the reno-protective effects of water intake may have been more evident.

Second, previous studies may have overlooked individual water requirements (water balance), which could explain the discrepancies. For example, both physical activity and body mass are positively associated with water intake^(3,6,49)^. However, although physical activity is negatively associated with CKD^(50)^, body mass is positively associated with CKD^(51)^. These factors may influence the relationship between water intake and CKD, even after adjustment for statistical analyses. To address this issue, water intake relative to an individual’s water requirements, after accounting for physical activity, body mass and other factors, should be assessed. Watanabe et al. showed that water deficiency has an L-shaped relationship with the risk of all-cause mortality and cardiovascular mortality^(17)^, similar to our results. Assessments of water deficit suggested that water intake excess or deficiency can be reasonably explained by considering an individual’s water requirements. Consequently, further prospective studies should assess the impact of water intake on renal protection and consider individual renal function and water requirements to validate our hypotheses.

Although the detailed mechanism by which water intake and water deficit are inversely associated with renal function is unknown, previous studies suggested two possibilities. First, low water intake induces hyperfiltration in glomeruli, which can have detrimental effects on the kidneys^(52)^. When the human body experiences low water intake or a low water volume, vasopressin secretion stimulates V2 receptors in the renal collecting ducts, and the osmotic driving force causes water reabsorption from liminal fluid, resulting in a marked decrease in the urinary flow rate. Vasopressin also activates the urea transporter UT-A1, which concentrates urea in urine^(53)^. Although this can inhibit water excretion, it also decreases urea excretion efficiency^(53,54)^ and causes hyperfiltration with renal enlargement^(55)^. Low water intake in humans increases the GFR^(56)^, and increased water intake decreases vasopressin secretion and GFR^(57)^. Second, dehydration may be indirectly associated with kidney function and affect health outcomes. Vasopressin is a risk factor for diabetic nephropathy and salt-sensitive hypertension^(58)^ because it stimulates sodium reabsorption through the epithelial sodium channel^(59)^. Additionally, increased levels of copeptin, which is an arginine vasopressin precursor, are associated with insulin resistance, metabolic syndrome^(60)^ and diabetes^(61)^. Thus, dehydration may be indirectly related to renal dysfunction because it adversely affects homeostasis of biological functions other than kidney function. However, our study found an inverse relationship between CKD prevalence and water deficit, even after adjusting for serum and urine osmolality, suggesting that water deficit may be associated with CKD prevalence independent of dehydration. Further basic and intervention studies are necessary to clarify the underlying mechanisms.

A strength of this study was that it explored the association between CKD and various factors such as urine, blood, total water intake and water deficit, which are crucial to assessing whether the relationship between total water intake and CKD is independently related to the dehydration status.

However, this study had some limitations. First, the cross-sectional design of the study made it impossible to assume that the observed associations between urinary and blood markers, total water intake and CKD prevalence were direct or temporal causal. Second, because the date of the survey and the region of residence of each participant were unavailable, the same altitude, temperature and relative humidity values were uniformly applied to all participants to calculate water turnover. A cross-sectional ecological study in Japan demonstrated that water turnover varies substantially by region and season^(62)^. The influence of these unmeasured environmental factors may have introduced measurement errors in the estimated water turnover. This kind of error in the exposure assessments might have weakened the relationship between CKD prevalence and water deficit. Third, self-reported 2-day dietary data may not have reflected an individual’s habitual water intake because of daily variations in dietary intake, recall bias and social desirability bias. Therefore, the absolute values of water intake and water deficit should be interpreted with caution. Finally, although we adjusted for several confounders, residual confounding of the water deficit and CKD by genetic and environmental factors may have occurred, thus limiting the generalisability of our results.

### Conclusions

We observed an L-shaped relationship between water deficit and CKD prevalence, independent of dehydration status. The OR for CKD prevalence plateaued at a water deficit of approximately –30 % to 0 %. Among the hydration markers, serum osmolality demonstrated the strongest association with CKD prevalence. Thus, assessing hydration status using serum osmolality, combined with estimating water turnover to establish individualised fluid intake targets, may contribute to the maintenance of kidney function. Nevertheless, given the limitations of the 24HDR method, water intake may have been underestimated, and the absolute value of the observed plateau should be interpreted with caution. Future prospective studies incorporating objective biomarkers are warranted to validate these findings and guide recommendations for water intake.

## Supporting information

Inoue et al. supplementary material Inoue et al. supplementary material

## Supplementary material

For supplementary material/s referred to in this article, please visit https://doi.org/10.1017/S0007114525105667
