# Supplementary material for: L-shaped relationship between water deficit and prevalence of chronic kidney disease among adults in the USA: National Health and Nutrition Examination Survey
Source: Br J Nutr. 2025 Nov 24;135(1):108–19. doi: 10.1017/S0007114525105667 (PMC12867604; doi:10.1017/S0007114525105667)
Supplement: Inoue et al. supplementary material [file S0007114525105667sup001.docx]

**Supplementary material**

L-shaped relationship between water deficit and prevalence of chronic kidney disease among adults in the United States: National Health and Nutrition Examination Survey

Yumiko Inoue, Daiki Watanabe, and Motohiko Miyachi

**SUPPLEMENTAL TABLE**

**Supplemental Table 1**. Correlations of water deficit with blood and urine indices

**Supplemental Table 2**. Odds ratios for chronic kidney disease according to water intake quartiles and blood and urine indices

**Supplementary Table 1** Correlations of water deficit with blood and urine indices

|  | Mean (SD) | Water deficit | |
| --- | --- | --- | --- |
|  |  | Spearman’s CC | Peason’s CC |
| **Total (*n* = 9332)** |  |  |  |
| Serum osmolarity (mOsm/kg) | 278(5) | -0.07* | -0.09* |
| BUN/Cr | 14.9(5.1) | -0.00 | -0.03* |
| Urine osmolality (mOsm/kg) | 629(267) | -0.19* | -0.19* |
| Urine flow rate (ml/min) | 1.0(1.0) | 0.23* | 0.20* |
| **Men (*n* = 4786)** |  |  |  |
| Serum osmolarity (mOsm/kg) | 279(5) | -0.11* | -0.13* |
| BUN/Cr | 13.7(4.3) | 0.01 | -0.02 |
| Urine osmolality (mOsm/kg) | 668(258) | -0.14* | -0.15* |
| Urine flow rate (ml/min) | 1.1(0.9) | 0.22* | 0.20* |
| **Women (*n* = 4546)** |  |  |  |
| Serum osmolarity (mOsm/kg) | 277(5) | -0.04* | -0.05* |
| BUN/Cr | 16.1(5.7) | 0.01 | -0.02 |
| Urine osmolality (mOsm/kg) | 589(269) | -0.26* | -0.25* |
| Urine flow rate (ml/min) | 1.0(1.0) | 0.24* | 0.20* |

BUN, blood urea nitrogen; CC, correlation coefficien; Cr, creatinine; SD, standard deviation.

^*^Indicates statistical significance of Spearman’s rank or Pearson’s correlation coefficient (*p* < 0.05).

**Supplementary Table 2** Odds ratios for chronic kidney disease according to water intake quartiles and blood and urine indices

|  | Case *n* (%) | Mean (SD) | Model 1^*^ | | Model 2^†^ | |
| --- | --- | --- | --- | --- | --- | --- |
|  |  |  | OR | 95% CI | OR | 95% CI |
| **Total water intake**^‡^ |  |  |  |  |  |  |
| Q1 (*n* = 2333) | 237 (10.2) | 1510 (309) | 1.00 | (Ref) | 1.00 | (Ref) |
| Q2 (*n* = 2333) | 164 (7.0) | 2238 (184) | 0.82 | (0.64-1.04) | 0.76 | (0.59–0.99) |
| Q3 (*n* = 2333) | 129 (5.5) | 2941 (232) | 0.77 | (0.59-1.01) | 0.72 | (0.54–0.96) |
| Q4 (*n* = 2333) | 58 (2.5) | 4505 (1203) | 0.55 | (0.38-0.78) | 0.57 | (0.39–0.82) |
| *p* for trend |  | | <0.001 | | <0.001 | |
| *p* for non-linearity |  |  | <0.001 | | <0.001 | |
| **Water turnover**^‡^ |  |  |  |  |  |  |
| Q1 (*n* = 2333) | 286 (12.3) | 2784 (206) | 1.00 | (Ref) | 1.00 | (Ref) |
| Q2 (*n* = 2333) | 131 (5.6) | 3154 (94) | 0.71 | (0.51-0.99) | 0.74 | (0.53–1.05) |
| Q3 (*n* = 2333) | 109 (4.7) | 3447 (73) | 0.94 | (0.62-1.44) | 1.11 | (0.71–1.73) |
| Q4 (*n* = 2333) | 62 (2.7) | 3777 (193) | 0.80 | (0.44-1.45) | 1.05 | (0.56–1.97) |
| *p* for trend |  | | <0.001 | | <0.001 | |
| *p* for non-linearity |  | | 0.002 | | <0.001 | |
| **Water deficit**^‡^ |  |  |  |  |  |  |
| Q1 (*n* = 2333) | 198 (8.5) | -52 (9) | 1.00 | (Ref) | 1.00 | (Ref) |
| Q2 (*n* = 2333) | 154 (6.6) | -31 (5) | 0.74 | (0.57-0.96) | 0.73 | (0.56–0.95) |
| Q3 (*n* = 2333) | 143 (6.1) | -11 (7) | 0.74 | (0.56-0.97) | 0.70 | (0.52–0.92) |
| Q4 (*n* = 2333) | 93 (4.0) | 32 (33) | 0.61 | (0.45-0.83) | 0.64 | (0.46–0.88) |
| *p* for trend |  | | 0.001 | | 0.001 | |
| *p* for non-linearity |  |  | <0.001 | | <0.001 | |
| **Serum osmolality**^‡^ |  |  |  |  |  |  |
| Q1 (*n* = 2662) | 40 (1.5) | 273 (3) | 1.00 | (Ref) | 1.00 | (Ref) |
| Q2 (*n* = 2499) | 85 (3.4) | 277 (1) | 1.97 | (1.31–2.96) | 1.95 | (1.29–2.94) |
| Q3 (*n* = 2179) | 110 (5.0) | 280 (1) | 2.02 | (1.37–2.99) | 1.98 | (1.33–2.93) |
| Q4 (*n* = 1992) | 353 (17.7) | 284 (3) | 4.56 | (3.19–6.54) | 4.46 | (3.10–6.41) |
| *p* for trend |  | | 0.001 | | <0.001 | |
| *p* for non-linearity |  |  | 0.900 | | 0.716 | |
| **Urine osmolality**^‡^ |  |  |  |  |  |  |
| Q1 (*n* = 2339) | 153 (6.5) | 270 (87) | 1.00 | (Ref) | 1.00 | (Ref) |
| Q2 (*n* = 2338) | 284 (12.1) | 540 (70) | 1.26 | (0.99–1.60) | 1.20 | (0.94–1.52) |
| Q3 (*n* = 2338) | 117 (5) | 750 (53) | 0.73 | (0.55–0.97) | 0.67 | (0.50–0.89) |
| Q4 (*n* = 2317) | 34 (1.5) | 96 1(87) | 0.60 | (0.39–0.91) | 0.52 | (0.35–0.79) |
| *p* for trend |  | | <0.001 | | <0.001 | |
| *p* for non-linearity |  |  | <0.001 | | <0.001 | |
| **Urine flow rate**^‡^ |  |  |  |  |  |  |
| Q1 (*n* = 2217) | 215 (9.7) | 0.3 (0.1) | 1.00 | (Ref) | 1.00 | (Ref) |
| Q2 (*n* = 2210) | 136 (6.2) | 0.6 (0.1) | 0.71 | (0.55–0.92) | 0.72 | (0.55–0.93) |
| Q3 (*n* = 2213) | 121 (5.5) | 1.0 (0.1) | 0.72 | (0.55–0.94) | 0.74 | (0.57–0.98) |
| Q4 (*n* = 2211) | 75 (3.4) | 2.2 (1.2) | 0.53 | (0.39–0.71) | 0.57 | (0.42–0.78) |
| *p* for trend |  | | <0.001 | | <0.001 | |
| *p* for non-linearity |  |  | <0.001 | | <0.001 | |

CI, confidence interval; OR, odds ratio; Q, quartile; Ref, reference; SD, standard deviation.

Data for participants with missing values were imputed by performing multiple imputation. Missing values were as follows: education (*n* = 10); smoking status (*n* = 262); alcohol consumption (*n* = 847); marital status (*n* = 472); poverty-to-income ratio (*n* = 778); medication use (*n* = 4); history of hypertension (*n* = 13); history of diabetes (*n* = 5); and physical activity (*n* = 29).

^*^Model 1: adjusted for body mass index, education, smoking status, alcohol consumption, marital status, poverty-to-income ratio, medication use, history of hypertension, history of diabetes mellitus, energy intake, and physical activity.

^†^Model 2: adjusted for all factors listed in model 1 as well as total water intake, water turnover, and water deficit were adjusted for serum and urine osmolality. The serum osmolality, urine osmolality, and urine flow rate were adjusted for total water intake and water turnover.

^‡^Total water intake quartiles are as follows: Q1, <1928; Q2, 1928–2572; Q3, 2573–3389; and Q4, ≥3390 mL/day. Water turnover quartiles are as follows: Q1, <2999; Q2, 2999–3312; Q3, 3313–3570; and Q4, ≥3571 mL/day. Water deficit quartiles are as follows: Q1, −39.7; Q2, −39.7 to −21.3; Q3, −21.4 to 1.2; and Q4, ≥1.3%. Serum osmolality quartiles are as follows: Q1, <276; Q2, 276–278; Q3, 279–281; and Q4, ≥282 mOsm/kg. Urine osmolality quartiles are as follows: Q1, <410; Q2, 410–655; Q3, 656–840; and Q4, ≥841 mOsm/kg. Urine flow rate quartiles are as follows: Q1, <0.5; Q2, 0.5–0.7; Q3, 0.8–1.2; and Q4, ≥1.3 mL/min.
